# Time-of-day dependent effects of midazolam administration on myocardial injury in non-cardiac surgery

**DOI:** 10.3389/fcvm.2022.982209

**Published:** 2022-10-28

**Authors:** Meghan Prin, Jack Pattee, David J. Douin, Benjamin K. Scott, Adit A. Ginde, Tobias Eckle

**Affiliations:** Department of Anesthesiology, University of Colorado Anschutz Medical Campus, Aurora, CO, United States

**Keywords:** circadian rhythms, midazolam, chronobiology, MINS, perioperative outcome, general anesthesia, MPOG, large dataset

## Abstract

**Background:**

Animal studies have shown that midazolam can increase vulnerability to cardiac ischemia, potentially *via* circadian-mediated mechanisms. We hypothesized that perioperative midazolam administration is associated with an increased incidence of myocardial injury in patients undergoing non-cardiac surgery (MINS) and that circadian biology may underlie this relationship.

**Methods:**

We analyzed intraoperative data from the Multicenter Perioperative Outcomes Group for the occurrence of MINS across 50 institutions from 2014 to 2019. The primary outcome was the occurrence of MINS. MINS was defined as having at least one troponin-I lab value ≥0.03 ng/ml from anesthesia start to 72 h after anesthesia end. To account for bias, propensity scores and inverse probability of treatment weighting were applied.

**Results:**

A total of 1,773,118 cases were available for analysis. Of these subjects, 951,345 (53.7%) received midazolam perioperatively, and 16,404 (0.93%) met criteria for perioperative MINS. There was no association between perioperative midazolam administration and risk of MINS in the study population as a whole (odds ratio (OR) 0.98, confidence interval (CI) [0.94, 1.01]). However, we found a strong association between midazolam administration and risk of MINS when surgery occurred overnight (OR 3.52, CI [3.10, 4.00]) or when surgery occurred in ASA 1 or 2 patients (OR 1.25, CI [1.13, 1.39]).

**Conclusion:**

Perioperative midazolam administration may not pose a significant risk for MINS occurrence. However, midazolam administration at night and in healthier patients could increase MINS, which warrants further clinical investigation with an emphasis on circadian biology.

## Introduction

In the perioperative setting for non-cardiac surgery, the incidence of myocardial injury in patients undergoing non-cardiac surgery (MINS) has been reported to be as high as 16% (1). Considering that >300 million surgeries are performed annually, and that changing demographics and evolving medical practices have resulted in an increasing number of surgical patients with elevated cardiovascular risk (2), these estimates are of paramount clinical significance (3). In fact, MINS is associated with substantial mortality. A recent single-center 10-year retrospective analysis reported 30-day mortality of 31% and 1-year mortality of 42% in patients who experienced a perioperative myocardial ischemia (MI) and underwent percutaneous revascularization after non-cardiac surgery (4). Strategies to reduce the incidence of perioperative myocardial ischemia and reperfusion injury are urgently needed (5).

Midazolam, a benzodiazepine which binds to receptor sites in the gamma-aminobutyric acid (GABA) system, first came into use in 1976 and is on the World Health Organization’s List of Essential Medicines. It is the most commonly used pre-procedural sedative-hypnotic worldwide (6), but it is also associated with significant clinical complications, which are most clearly described in the critical care literature. A growing body of evidence shows that benzodiazepines are associated with poor patient outcomes including delirium, duration of mechanical ventilation, and ICU length of stay (7). Benzodiazepine infusions, as compared to propofol, are also linked to an increased likelihood of death among patients who receive mechanical ventilation (8). Because of its adverse associations, midazolam is no longer recommended as a first-line sedative on critical care units (9–12). Whether midazolam use prior to surgical procedures is similarly associated with adverse outcomes is unknown.

Recently, our group determined that the circadian rhythm protein Period 2 (PER2) provides robust cardio-protection from myocardial ischemia (MI) in an animal model (13, 14). We also demonstrated that midazolam increases vulnerability to cardiac ischemia by downregulating PER2 (15). Because perioperative MI is the most common perioperative cardiovascular complication and sedative-hypnotics can alter the expression of PER2, a sedative-mediated downregulation of PER2 could be detrimental if myocardial ischemia and reperfusion occurs (16–18). As a first effort in studying this association in humans, we hypothesized that midazolam administration during the perioperative period would be associated with an increased incidence of MINS and that this relationship depends on the 24-h cycle of human circadian physiology.

## Materials and methods

### Approvals

Approval was obtained, and waiver of written informed consent was granted from the Institutional Review Board [Colorado Multiple Institutional Review Board (COMIRB)] at the University of Colorado Denver, USA (#09-0674). In keeping with the Multicenter Perioperative Outcomes Group Bylaws at the University of Michigan, this study protocol was presented to the Multicenter Perioperative Outcomes Group Perioperative Clinical Research Committee and was approved on August 12, 2019. We followed the Strengthening the Reporting of Observational Studies in Epidemiology (STROBE) checklist in developing this manuscript.

### Data source and study inclusion and exclusion criteria

The Multicenter Perioperative Outcomes Group (MPOG) database, as well as methods for data entry, validation, and quality assurance, have been previously described (19) and have been used for multiple published observational studies (20, 21). MPOG data are drawn from cases documented in the Electronic Health Record at participating sites. These data are extracted, standardized, joined to additional laboratory, billing, and diagnosis coding data, and de-identified except for date of service, producing a limited dataset.

Data for 2,740,183 subjects undergoing non-cardiac surgical procedures between 1 January 2014, and 31 December 2019, were obtained from MPOG. Various preoperative demographic and comorbidity variables were available, as were data on the timing and dosage of midazolam administrations and the timing and magnitude of troponin-I lab values. From this sample of 2,740,183 subjects, we excluded all subjects who met one or more of the following exclusion criteria: emergent cases, outpatient procedures, patients admitted for less than 24 h, patients with ASA value 5 or 6, preoperatively intubated patients, patients with preoperative troponin elevation (defined as a recorded troponin I value ≥0.01 ng/ml within 42 days of the start of anesthesia), pre-induction vasopressor or inotrope infusion, intraoperative transfusion >4 units of blood, estimated blood loss >2000 ml, lung-transplant surgery (CPT code between 32850 and 32856), liver-transplant surgery (CPT code between 47133 and 47147), cardiac procedures (CPT code between 33016 and 33999), and individuals under 18 years of age. 2,264,900 subjects remained after applying these exclusion criteria.

Perioperative MINS was defined as having at least one troponin-I lab value ≥0.03 ng/ml in the period from anesthesia start to 72 h after anesthesia end. Perioperative midazolam administration was defined as having at least one documented midazolam administration in the period from 2 h before anesthesia start to anesthesia end. The mean dose of midazolam was 2.3 mg.

As typical for large observational data extracted from electronic health records, there was some amount of missingness in our data. Of those 2,264,900 subjects who met the inclusion criteria, 9.76% had missing information regarding BMI and 0.05% regarding sex. We opted to only include those subjects without missingness in our analytic dataset. Given that our sample size was substantial, we were not concerned with efficiency loss due to a complete case analysis as opposed to an imputation approach. Recommendations from literature indicate that, as long as there is no missingness in an effect modifier variable (midazolam administration, time of day, or ASA class), complete case analysis will be unbiased regardless of the missingness pattern of the covariate (i.e., missing completely at random, missing at random, or not missing at random) (22). In this case, as BMI was not an effect modifier in our analysis, a complete case analysis will not bias our inference. Our final analytic dataset consisted of 1,773,118 subjects. Of these subjects, 16,404 (0.93%) experienced perioperative MINS, and 951,345 (53.7%) had a perioperative administration of midazolam (mean dose 2.3 mg).

### Patient variables

**FIGURE 1:**
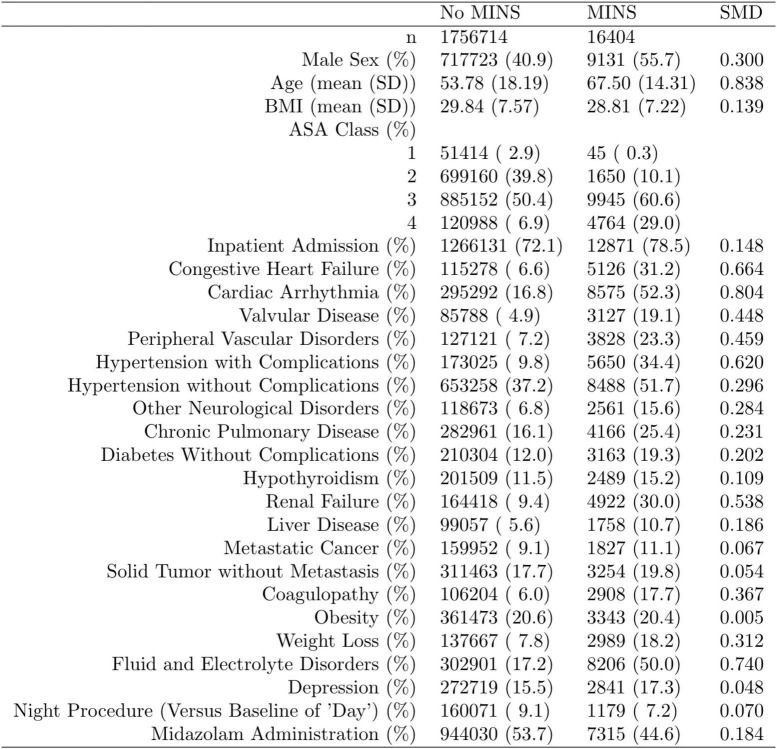
Distribution of the propensity score stratified by whether subjects had perioperative myocardial injury in non-cardiac surgery (MINS). Standardized mean difference (SMD) column represents the standardized mean difference.

Complete data from the Multicenter Perioperative Outcomes Group database included age, sex, body mass, institution, American Society of Anesthesiologists (ASA; Schaumburg, Illinois) physical status and various comorbidities (Figure 1).

### Outcomes of interest

The primary outcome of our study was perioperative MINS in relation to human circadian physiology.

### Statistical analysis

Data are presented as coefficient estimates (adjusted odds ratios) with confidence intervals. To account for possible bias and confounders in the observational data, propensity scores were used to balance the distribution of baseline covariates between the population with and without perioperative midazolam administration (23, 24). Specifically, the method of inverse probability of treatment weighting was used. The propensity score and the associated inverse probability of treatment weights were estimated in R *via* the “WeightIt” package (version 0.12.0). The propensity score was estimated *via* logistic regression using all baseline covariates as predictors, with no higher-order or interaction effects. A recounting of the baseline covariates and their stratified distribution in the analytic dataset is presented in Figure 1. All covariates listed in Figure 1 were included as predictors in the propensity-score model. Whether or not those covariates listed in Figure 1 are technically considered confounders or not, however, is immaterial (25). Nevertheless, we note here that their inclusion in the propensity score model and the subsequent reweighting procedure precludes any potential confounding effect of these variables on our inferences of interest. Comorbidities with <5% prevalence were excluded from Figure 1 and the ensuing propensity-score model. Only covariates that were measured before the administration of midazolam were included in the propensity score model, following established practice (26). This precluded the inclusion of perioperative variables such as intraoperative blood pressure. Balance diagnostics were assessed *via* the “WeightIt” package, and balance was assessed as standardized mean difference <0.1 (25).

For assessing the effect of (binary) midazolam administration on perioperative MINS, we used a weighted logistic regression model with robust variance estimation, implemented in R *via* the “survey” package (version 4.1-1). For this analysis, stabilized inverse probability of treatment weights were used. Circadian physiology analyses, including assessment of potential effect modification of time of day or ASA class on the effect of midazolam administration on perioperative MINS, were conducted using marginal structural models with an interaction effect. The time of day was divided into daytime (between 6:00 and 18:00) and a nighttime period (between 18:00 and 06:00) based on anesthesia start. Marginal structural models were also estimated *via* weighted logistic regression with robust variance estimation *via* the R package “survey.” For theses analyses *via* marginal structural models, weights were stabilized as per Section 12.5 of Hernán and Robins (27). This stabilization entails using the ratio of the probability of midazolam administration predicted by the modifier (e.g., time of day or ASA class) to the probability of midazolam administration predicted by all baseline covariates as weights in the marginal structural model. The form of the inverse probability of treatment weights was different for the analysis of the effect of midazolam administration on perioperative MINS and each of the analyses assessing the potential effect modification of time of day or ASA class on the effect of midazolam administration on perioperative MINS.

Per Hernán and Robins, whether the effect of exposure α on the outcome is modified by the particular covariate *V* while adjusting for the set of baseline covariates *L*, the following model is used: *E*[*Y*|α,*V*] β_0_β_1_αβ_2_*V*αβ_3_*V* [1]. In order to isolate the causal effect of α (in our context, midazolam administration) on a binary outcome (in our context, MINS) while assessing potential effect modification by *V* (in our context, either time of day or ASA class), we estimated the above model in a weighted logistic regression framework *via* the R package survey (version 4.1-1), where weights are generated *via* propensity scoring. Weights are estimated as follows, per the recommendation of Hernán and Robins:


$$S\,W^{A}\,\left(V\right)=\frac{f\,\left[\alpha |V\right]}{f\,\left[\alpha |L\right]}\,\left[2\right]$$

We estimated two models in this framework: one to assess whether ASA class modifies the effect of midazolam administration on MINS, and one to assess whether time of day modifies the effect of midazolam administration on MINS. For the ASA model, *V* is the ASA class (either high: ASA 3 or 4, or ASA low: 1 or 2). For the time-of-day model, *V* is the time of day (either overnight or day). Thus, for each of these two paradigms, we estimated model [1] *via* weighted logistic regression using weights as described in [2]. For the ASA model, weights as described in [2] are equal to the ratio of the probability of midazolam administration (α) as predicted by ASA class (*V*) to the probability of midazolam administration as predicted by all baseline covariates including ASA class (*L*). For the time-of-day model, weights as described in [2] are equal to the ratio of the probability of midazolam administration (α) as predicted by time of day (*V*) to the probability of midazolam administration as predicted by all baseline covariates including time of day (*L*).

### Power analysis

The large sample size (>1,400,000 non-cardiac patients) and an estimated incidence of 0.4% for MINS provided a >99% power at an alpha of 0.05 to detect any differences between the groups receiving midazolam or not receiving midazolam.

## Results

### Study populations and outcomes

**FIGURE 2:**
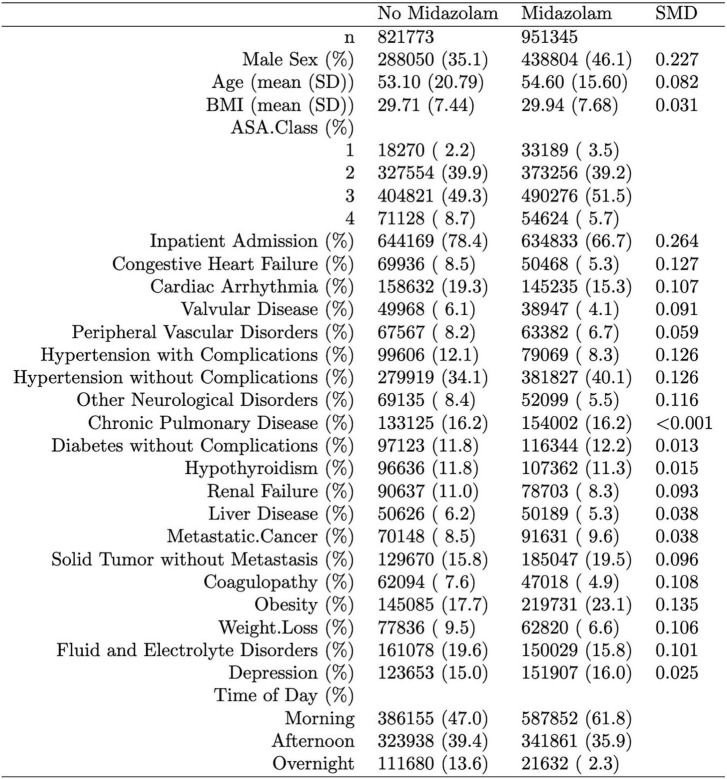
Distribution of the propensity score stratified by whether subjects had perioperative midazolam. “SMD” column represents the standardized mean difference. The percentage of patients with a neuromuscular block only were 0.4% in the midazolam group and 1.6% in the non-midazolam group. The lab cTnI (ng/mL), used to define our myocardial injury in non-cardiac surgery (MINS) endpoint, was present in 4.7% of subjects in both the group with and without midazolam.

**FIGURE 3:**
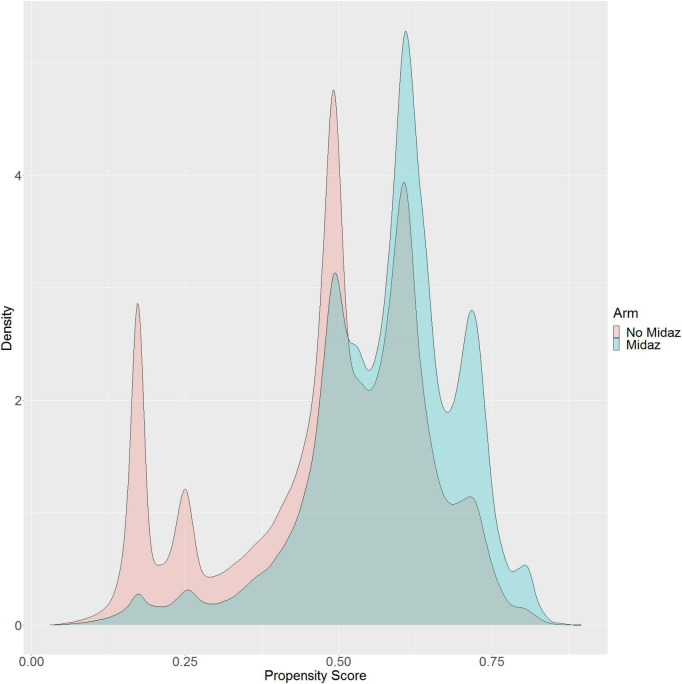
Distribution of the propensity score stratified by whether subjects received perioperative midazolam.

**FIGURE 4:**
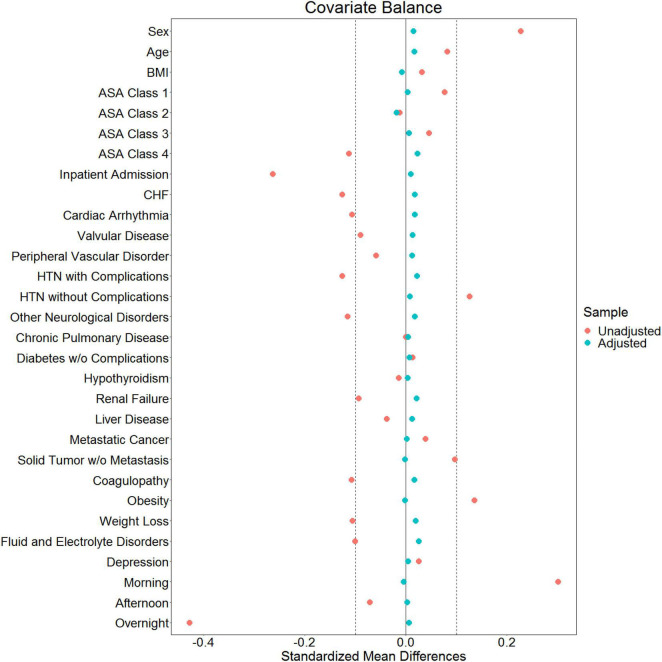
Love plot showing baseline imbalances for the covariates of interest and the effectiveness of balancing *via* the inverse probability of treatment weights.

Of 2,264,900 cases that were eligible for analysis, 491,782 were excluded for missing data. A total of 1,773,118 cases from 50 institutions were available. Figures 1, 2 describe the distribution of all baseline covariates in the study stratified by whether perioperative MINS occurred, or weather Midazolam was administered, respectively. The unadjusted rate of perioperative MINS in subjects with perioperative midazolam was 7,315 [0.77%], and the rate of MINS in subjects without perioperative midazolam was 9,089 [1.1%]. However, midazolam administration was associated with baseline covariates which indicated that these variables could be confounders. This association is displayed in Figure 3, which shows propensity scores stratified by whether subjects received perioperative midazolam. Therefore, propensity scores were used to balance the distribution of baseline covariates between the population with and without perioperative midazolam administration. Weighting the samples by the inverse probability of treatments successfully accounted for imbalances in the baseline covariates as illustrated in Figure 4. Following propensity score correction, on average, no association between perioperative midazolam administration and the rate of MINS was observed (OR 0.98, CI [0.94, 1.01]).

Based on observations that midazolam alters the risk for myocardial ischemia in a circadian-rhythm-dependent manner, we performed two circadian physiology related analyses: (1) time-of-day dependent effects of midazolam administration on MINS risk, as circadian mechanisms are expected to cause variations in MINS risk throughout a full circadian cycle (24 h); and (2) ASA classification dependent effects of midazolam administration on MINS risk, as higher ASA classification (3+4) include an older population with more comorbidities in whom circadian rhythms are known to fade and become dysfunctional (28, 29).

As shown in Table 1A the baseline MINS risks significantly varied throughout the day. There was a significantly decreased baseline risk of MINS during overnight when compared to daytime surgeries (OR: 0.52, 95% CI [0.48, 0.56]).

**TABLE 1:** Effect of circadian physiology on MINS.

**(A) Time of day effect on MINS**		
*No midazolam*	Odds ratio	95% Confidence interval
MINS–overnight versus daytime surgery	0.52	[0.48, 0.56]
**(B) Time of day effect of midazolam on MINS**		
*Midazolam* vs. *no midazolam*		
MINS during daytime surgery	0.84	[0.80, 0.87]
MINS during overnight surgery	3.52	[3.10, 4.00]
**(C) ASA effect only on MINS**		
*No midazolam*		
MINS in ASA 3+4 vs. ASA 1+2	7.42	[6.84, 8.04]
**(D) MINS in different ASA classes**		
*Midazolam* vs. *no midazolam*		
MINS in ASA 1+2	1.25	[1.13, 1.39]
MINS in ASA 3+4	0.94	[0.91, 0.98]
**(E) Time of day effect of midazolam on MINS in ASA 1+2**		
*Midazolam* vs. *no midazolam*		
MINS during daytime surgery	1.11	[0.998, 0.123]
MINS during overnight surgery	3.59	[2.4, 5.38]
**(F) Time of day effect of midazolam on MINS in ASA 3+4**		
*Midazolam* vs. *no midazolam*		
MINS during daytime surgery	0.85	[0.82, 0.88]
MINS during overnight surgery	1.67	[1.46, 1.91]

(A,B) Coefficient estimates and confidence intervals for the marginal structural model assessing the effect modification of time of day on midazolam administration. (C,D) Coefficient estimates and confidence intervals for the marginal structural model assessing the effect modification of the American Society of Anesthesiologists (ASA) class on midazolam administration. (E,F) Coefficient estimates and confidence intervals for the marginal structural model assessing the effect modification of time of day on midazolam administration in ASA 1+2 or ASA 3+4 patients.

The time-of-day dependent effect of midazolam administration on MINS occurrence is shown in Table 1B. The interaction between time-of-day and midazolam administration was significant (*p* 2 10^−16^), demonstrating that the effect of midazolam on MINS occurrence was modified by the time-of-day. While midazolam administration during the daytime decreased the risk for MINS (OR 0.84, 95% CI [0.80, 0.87], the administration of midazolam to individuals who underwent overnight surgery significantly increased the risk for MINS (odds ratio of 3.52 for MINS, 95% CI [3.10, 4.00]), when compared to no midazolam administration. Our nighttime analysis included 161,250 individuals (Figure 1).

Next, we evaluated the effect of the ASA classification on the MINS baseline risk without any midazolam administration. As expected, patients in the ASA 3+4 class had a significantly higher MINS risk than patients in the ASA class 1+2 (OR 7.42, 95% CI [6.84, 8.04], Table 1C).

Finally, we analyzed the relationship between midazolam administration and MINS risk in ASA 1+2 and ASA 3+4 patients (Table 1D). We found a significant interaction between the ASA class and midazolam administration (*p* 2.8 10^−7^), demonstrating that the effect of midazolam on MINS occurrence was modified by the ASA class. Indeed, midazolam was associated with a moderately increased rate of MINS in the low ASA (1+2) class group (OR: 1.25, 95% CI [1.13, 1.39]) who had a total MINS rate of 0.23%. However, the association between midazolam and MINS was substantially attenuated, and in fact changed directions, when a subject was in the high ASA group (3+4), who had a total MINS rate of 1.44%. For a subject in the high ASA group, the administration of midazolam was associated with an odds ratio of 0.94 for MINS (95% CI [0.91, 0.98]).

However, as shown in Table 1E,F, the administration of midazolam during overnight surgeries significantly increased the risk for MINS in ASA 1+2 as well as in ASA 3+4 patients (odds ratio of 3.59 for MINS, 95% CI [2.4, 45.38] for ASA 1+2 and odds ratio of 1.67 for MINS, 95% CI [1.46, 1.91] for ASA 3+4), when compared to no midazolam administration.

## Discussion

In this study, we examined the relationship between perioperative midazolam administration and MINS. Overall, we did not find an association between midazolam administration and the rate of MINS. However, midazolam administration was associated with an increased risk of MINS when surgeries occurred at night or in healthier patients in the ASA 1+2 class. Interestingly, we found a MINS risk reduction when midazolam was given to higher risk patients (ASA 3+4 class). When assessing the time of day, however, the increased risk of MINS at night was present in both ASA 1+2 and ASA 3+4.

**FIGURE 5:**
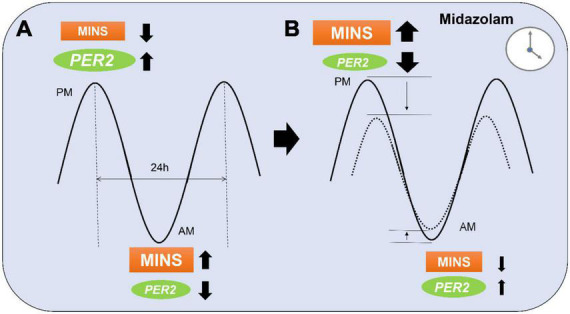
Proposed model of midazolam modifying myocardial injury in non-cardiac surgery (MINS) risk during a 24 h period. (A) At the peak of PER2 in the evening (p.m.), MINS occurs less frequent than during the PER2 trough in the morning (a.m.). (B) Midazolam administration at night might lower the PER2 peak which results in a higher occurrence of MINS. Midazolam administration during the daytime might have opposite effects and lower the PER2 trough resulting in less MINS occurrence. Dotted line indicates MINS risk in patients with midazolam.

The endogenous circadian clock mechanism involves a cell autonomous transcription–translation feedback loop. During the day, the transcription factor CLOCK interacts with BMAL1 to activate transcription of the *Per* and Cryptochrome (*Cry*) genes, resulting in high levels of these transcripts. The resulting PER and CRY proteins translocate to the nucleus to inhibit their own transcription (30). The entire cycle takes approximately 24 h. This process is also active without external cues and takes approximately 25 h in humans (31). Besides autonomous mechanisms, resetting of the circadian clock by photic induction of *Per1* and *Per2* genes is mediated by the binding of phosphorylated CREB (cyclic AMP responsive element-binding protein) to a cAMP-responsive element (CRE) in the respective promoters (32). Since the circadian rhythm in humans is dominantly regulated by sunlight (33), circadian proteins cycle over a 24-h period, reaching their peak expression at night. The incidence of myocardial injury has been found to be lower at night in both mice and humans with an abrupt increase in the early morning hours (6 a.m.) (14). Mouse studies have revealed a reciprocal relationship between myocardial injury and PER2 protein expression (13). In-depth genetic studies in mice have recently identified endothelial PER2 as an endogenous cardioprotective mechanism (14). In keeping with these findings, we found a lower baseline risk for MINS during the night when compared to the day. We also found a correlation between the time of midazolam administration and the risk of MINS. This would support data from animal studies showing a link between midazolam, circadian protein expression, and ischemia, and suggests that midazolam also interferes with the circadian system in humans. Midazolam increases GABA_*A*_ signaling, an important component of circadian rhythm protein regulation (34). In fact, GABA_*A*_ activation can inhibit the expression of circadian *Per2* mRNA (35). As midazolam could lower the naturally occurring higher nighttime-PER2 levels, it is plausible that the heart could be more susceptible to injury when midazolam is given at night compared to the day. Further, as we also found that midazolam decreased the risk of MINS during the daytime, midazolam might also cause a reduction of the amplitude not only at the peak but also at the trough (Figure 5).

The relationship between ASA class and risk of MINS after midazolam exposure may have a similar physiologic basis. Because most medical comorbidities increase in prevalence with age, ASA classes 3 and 4 presumably include an older patient population. Indeed, our ASA 1+2 had a mean age of 45.7 and our ASA 3+4 group had a mean age of 59.9 years. In older adults, the amplitude and peak expression of circadian rhythms decreases by 20 to 40% (36). In addition, animal studies have shown that aging results in the diurnal rhythm amplitude of PER2 (37). Exposures that interfere with circadian protein expression might therefore be more harmful in younger patients who are accustomed to a higher circadian amplitude and baseline expression of circadian proteins. Indeed, we identified a significant increase of MINS in the ASA 1+2 group when midazolam was given perioperatively. Surprisingly, we found a possible protective effect of midazolam in the ASA 3+4 group. Although small, this reduction in risk is harder to explain *via* circadian mechanisms. One possibility is that administration in patients with many comorbidities resulted in lower cumulative anesthetic doses, reduced stress responses, and more hemodynamic stability during surgery which ultimately could have resulted in fewer cardiac complications. Analyzing ASA 5 and 6 patients could potentially have given more insight into this phenomenon. However, since we excluded ASA 5 and ASA 6 patients due to their emergent and complex status, this will require future evaluation. Regardless, when we considered the time of day, an increased risk of MINS at night was present in both ASA 1+2 and ASA 3+4, suggesting that the time of day had a higher impact on the occurrence of MINS.

### Limitations

A limitation of our current study is the retrospective analysis of administrative data which shows associations among variables but not necessarily causal relationships, a common problem of unmeasured confounding. However, propensity scores were used to balance the distribution of baseline covariates between the population with and without perioperative midazolam administration which has been shown to contribute to a more precise estimation of the treatment response (38).

Further, perioperative midazolam could have been administered for a variety of indications other than anxiolysis, including in hemodynamically unstable patients to minimize the use of inhaled agents or more vasodilatory or cardioactive sedative-hypnotics. However, this is likely a small subset of our population and would not explain our finding that midazolam has a time-of-day dependent effect on the rate of MINS.

In addition, other causes of MINS (occurring in the 72-h period after surgery) such as from postoperative ischemia, sepsis, neurogenic cardiomyopathy, etc., might have been missed and could therefore be possible confounders. Again, this would not fully account for the findings of our analyses regarding time-of-day or ASA-class effects.

Finally, MINS was most likely underestimated in our sample as most institutions do not measure postoperative troponin routinely. As a result, our analysis treated any subject without a perioperative troponin-I as negative for perioperative MINS resulting in a MINS rate of 0.93%. However, previous studies have reported a MINS rate of 8.0% among patients that are 45 years or older (39). Given this data, undetected MINS almost certainly occurred in our cohort, but it seems reasonable to assume that undetected MINS was proportionate in individuals with and without midazolam administration.

## Conclusion

This large retrospective observational study suggests that perioperative administration of midazolam during nighttime surgeries as well as administration in healthier patients may increase the risk of MINS. The fact that we did not find an overall relationship between perioperative midazolam and MINS but found differences in risk at certain times of day suggests that chronobiology may play an important role in MINS and other perioperative outcomes. Future studies are needed that assess these time-of-day dependent effects.

## Data availability statement

The original contributions presented in this study are included in the article/supplementary material, further inquiries can be directed to the corresponding author.

## Ethics statement

The studies involving human participants were reviewed and approved by Colorado Multiple Institutional Review Board (COMIRB) at the University of Colorado Denver, United States (#09-0674). Written informed consent for participation was not required for this study in accordance with the national legislation and the institutional requirements.

## Author contributions

MP, JP, DD, BS, and AG: wrote the manuscript and analyzed the data. TE: designed the study, wrote the manuscript, and analyzed the data. All authors contributed to the article and approved the submitted version.
